# Red fluorescent cAMP indicator with increased affinity and expanded dynamic range

**DOI:** 10.1038/s41598-018-20251-1

**Published:** 2018-01-30

**Authors:** Yusaku Ohta, Toshiaki Furuta, Takeharu Nagai, Kazuki Horikawa

**Affiliations:** 1 Department of Optical Imaging, The Institute of Biomedical Sciences, Tokushima University Graduate School, 3-18-15 Kuramoto-cho, Tokushima City, Tokushima 770-8503 Japan; 2 Department of Biomolecular Science, Toho University, 2-2-1 Miyama, Funabashi, 274-8510 Japan; 3 Department of Biomolecular Science and Engineering, The Institute of Scientific and Industrial Research, Osaka University, Mihogaoka 8-1, Ibaraki, Osaka 567-0047 Japan

## Abstract

cAMP is one of the most important second messengers in biological processes. Cellular dynamics of cAMP have been investigated using a series of fluorescent indicators; however, their sensitivity was sub-optimal for detecting cAMP dynamics at a low concentration range, due to a low ligand affinity and/or poor dynamic range. Seeking an indicator with improved detection sensitivity, we performed insertion screening of circularly permuted mApple, a red fluorescent protein, into the cAMP-binding motif of PKA regulatory subunit Iα and developed an improved cAMP indicator named R-FlincA (Red Fluorescent indicator for cAMP). Its increased affinity (*K*_d_ = 0.3 μM) and expanded dynamic range (860% at pH 7.2) allowed the detection of subtle changes in the cellular cAMP dynamics at sub-μM concentrations, which could not be easily observed with existing indicators. Increased detection sensitivity also strengthened the advantages of using R-FlincA as a red fluorescent indicator, as it permits a series of applications, including multi-channel/function imaging of multiple second messengers and combinatorial imaging with photo-manipulation. These results strongly suggest that R-FlincA is a promising tool that accelerates cAMP research by revealing unobserved cAMP dynamics at a low concentration range.

## Introduction

3′,5′-cyclic adenosine monophosphate (cAMP) is one of the major signalling mediators that regulates a variety of cellular functions, including synaptic plasticity of neurons^1^, T cell immune response^2^, insulin secretion from pancreatic β-cells^3^, and excitation–contraction coupling in cardiac myocytes^4^. As in the case of Ca^2+^ imaging^5–12^, spatio-temporal dynamics of cellular cAMP have been investigated with the help of a variety of fluorescent cAMP indicators^13,14^. FlCRhR^15^, the first developed Förster resonance energy transfer (FRET)-based indicator, can monitor cAMP dynamics through intermolecular dissociation of fluorescently-labelled Protein kinase A (PKA) subunits. Limitations in the cell loading of this semi-synthetic dye have been overcome by genetically encoded FRET-based indicators (Supplementary Tables S1 and S2^13^) that incorporate cAMP-binding proteins, such as PKA^16–19^ and Exchange Protein directly Activated by cAMP^20,21^ (EPAC). Although ratiometric and life-time observations of FRET-based indicators allowed for robust quantification of cAMP dynamics, the changes in their signal intensity were not prominent^16–21^. Changes in signal intensities of these indicators have been partly increased through optimization of FRET parameters (*e*.*g*. donor-acceptor configuration^22–25^). Alternatively, the single fluorescent protein (1-FP)-based indicators showing the large intensity changes have been developed. Flamindo2^26^ (FL2), a yellow fluorescent indicator consisting of Citrine (YFP variant) inserted with EPAC, was reported to display an increased dynamic range (D.R., ~300%) that facilitated the detection of an artificially induced cAMP response by a strong stimuli, such as Forskolin (FSK). Pink Flamindo^27^ (Pink-FL), a red colour variant of FL2 consisting of mApple, allowed advanced applications including *in vivo* imaging and optogenetic manipulations. However, it was difficult to observe physiological cAMP dynamics coupled with spontaneous cellular activities in *in vitro* and *in vivo*. As the concentration range of spontaneous cAMP dynamics are expected to be low^25,28,29^ (a few tens of nM to a few μM), sensitive indicators with both a high affinity and large D.R. would be valuable than low-affinity indicators (*K*_d_s of 3.2 μM and 7.2 μM for FL2^26^ and Pink-FL^27^, respectively). The affinity of 1-FP based indicator for cAMP can be increased by utilizing a high-affinity cAMP-binding motif of PKA regulatory subunits^13,30^, rather than utilizing a low-affinity EPAC sequence^13,26,27,31^. To increase the D.R., hopefully with brightness being another important parameter determining the performance of 1-FP indicators, testing the different molecular design from FL2 and Pink-FL (*i*.*e*., insertion of the sensor motif into a FP reporter) might be effective, since an alternative design that worked for high-performance Ca^2+^ indicators like GCaMPs^9,10,12^/pericams^7^/GECOs^11^ (*i*.*e*., the insertion of an FP reporter into the sensor motif), has not been examined for a cAMP indicator thus far.

To develop an improved cAMP indicator, we tested the previously unexamined design of a 1-FP cAMP indicator, in which cp146mApple, circular permuted red fluorescent protein^11,32^, was inserted into the high-affinity cAMP-binding motif of PKA regulatory subunit^30^. With a minimal screening effort, we successfully developed an improved cAMP indicator, termed R-FlincA. Its increased affinity (*K*_d_ = 0.3 μM) and expanded D.R. (860% at pH 7.2) allowed for high-sensitivity detection of faint cAMP dynamics associated with spontaneous cellular signalling, which could not be easily achieved by other 1-FP cAMP indicators. Increased detection sensitivity also strengthened the advantages of R-FlincA as a red fluorescent indicator, as it provided new insights regarding the cAMP dynamics in the insulin secretion of pancreatic β-cells and in the development of *Dictyostelium discoideum* cells by multi-channel/function imaging with spectrally distinct (blue to yellow) indicators. These results suggest that R-FlincA is a promising tool for the acceleration of cAMP research.

## Results

### Generation of an improved red indicator for cAMP

**Figure 1:**
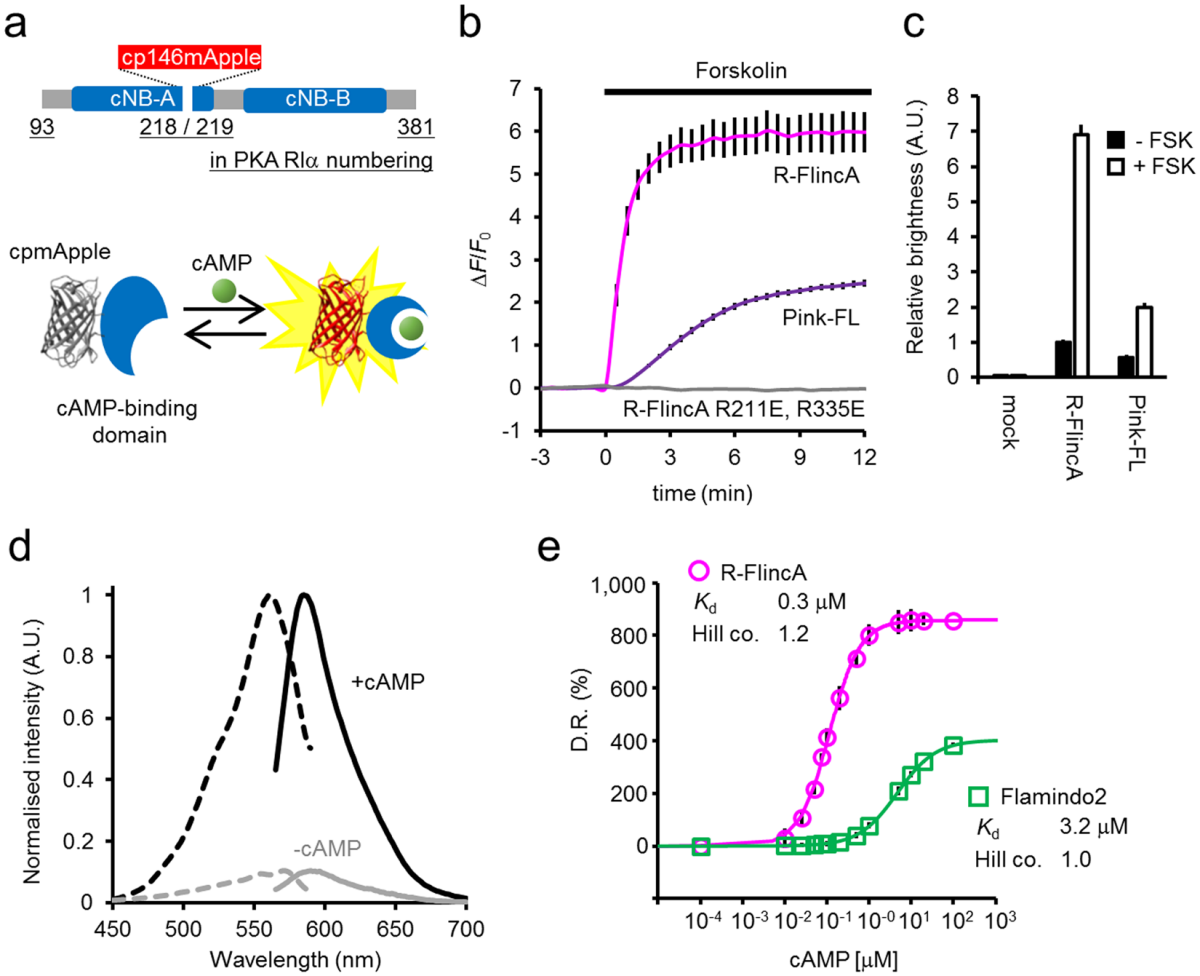
Novel red cAMP indicator with increased affinity and expanded dynamic range. (**a**) Molecular design of R-FlincA. The conformational change of PKA RIα upon cAMP-binding increases the fluorescence of cpmApple. (**b**) The fluorescence signal change of R-FlincA, its cAMP insensitive variant (R211E and R335E), and Pink-FL. 293 T cells expressing the indicator were stimulated by FSK (50 μM) to elevate cellular cAMP. Δ*F*/*F*_0_ for R-FlincA and Pink-FL are 6.04 ± 0.52 (mean ± SEM) and 2.51 ± 0.09, respectively. Bars represent ± SEM in three independent experiments (N = 30, 30 and 40 cells for R-FlincA, mutated R-FlincA and Pink-FL). (**c**) The in-cell brightness of cAMP indicators. Shown were the intensity of red fluorescence divided by that of ECFP co-expressed as a reference marker using P2A system. Values of mock control (ECFP only) before and after FSK-stimulation were 0.05 ± 0.01 (mean ± SEM) and 0.06 ± 0.01; 1.00 ± 0.06 and 6.91 ± 0.28 for R-FlincA; 0.57 ± 0.06 and 2.00 ± 0.11 for Pink-FL, respectively. Bars represent + SEM (N = 30, 30 and 40 cells for mock, R-FlincA and Pink-FL, respectively). See also Supplementary Fig. S4. (**d**) Excitation (dashed line) and emission (solid line) spectra of R-FlincA at pH 7.2 for free and saturated cAMP, normalised by the the values of cAMP-bound form. The average of three independent experiments were shown. (**e**) Titration curves for cAMP (log scale) of R-FlincA and Flamindo2 at pH 7.2. For R-FlincA and Flamindo2, *K*_d_ = 0.30 ± 0.02 and 3.19 ± 0.06 μM and Hill coefficient = 1.19 ± 0.06 and 0.96 ± 0.09, respectively. Bars represent ± SEM of three independent experiments. See also Supplementary Fig. S3a.

To develop an improved 1-FP cAMP indicator with a high affinity and expanded D.R., we employed a previously unexamined strategy, *i*.*e*., the insertion screening of cp146mApple (derived from R-GECO1.2^32^) into a high-affinity cAMP-binding motif from the human PKA regulatory subunit Iα (RIα). In total, 13 different positions in the long and short forms of the cAMP-binding motifs of RIα (a.a.93–381 or a.a.93–246) were tested in the cell-based assay to measure the signal change in response to FSK stimulation. As a result, RIα A + B 218, in which cp146mApple was inserted in one of the core cAMP-binding motifs (cNB-A), displayed the largest increase in fluorescence intensity (Δ*F*/*F*_0_, 600%, Fig. 1b and Supplementary Fig. S1), which was 2.4-times larger than that of Pink-FL^27^ (Δ*F*/*F*_0_, 250%, Fig. 1b), and was designated as R-FlincA (Red Fluorescent indicator for cAMP). In general, the brightness of the 1-FP indicator is an important determinant of its performance, and high D.R. is not necessarily useful if brightness is diminishingly low within the cell. To assess this possibility, we expressed the indicator together with a reference fluorescence marker (ECFP) using a bi-cistronic expression system. In the resting state, the fluorescence intensity of R-FlincA normalised with that of ECFP was comparable to that of Pink-FL, and the both were significantly higher than that of the mock control (>10-fold, Fig. 1c). When stimulated by FSK, cells expressing R-FlincA showed 3.5-fold higher fluorescence intensity level than Pink-FL, indicating that R-FlincA reports FSK-induced cAMP dynamics with a higher brightness than Pink-FL (Fig. 1c). R-FlincA mutated with cNB-motifs (R211E and R335E in RIα numbering^33^) exhibited no change in the fluorescence intensity upon FSK-stimulation (Fig. 1b), suggesting that the observed signal change would be dependent on cAMP-binding.

### Increased affinity and expanded dynamic range of R-FlincA

*In vitro* properties of R-FlincA were characterized using the purified recombinant protein. R-FlincA showed bimodal peaks in both excitation (571 nm and 561 nm) and emission wavelengths (590 and 585 nm) in the absence or presence of cAMP, respectively (Fig. 1d). The D.R. of R-FlincA was 860% at pH 7.2 (Fig. 1d), which is the highest among existing cAMP indicators (Supplementary Tables S1 and S2^13^). The apparent *K*_d_ for cAMP was 0.3 μM (pH 7.2, Fig. 1e), being ~11-fold higher affinity than FL2^26^ (*K*_d_, 3.2 μM). An observed Hill coefficient of 1.2 (pH 7.2, Fig. 1e) suggested low cooperativity between the two cAMP-binding sites. Indeed, a single mutation in the cNB-A domain (R211E in RIα numbering) completely abolished the fluorescence increase in response to cAMP, while a single mutation in the cNB-B domain (R335E in RIα numbering) had negligible effects on the D.R., affinity, and cooperativity values (Supplementary Fig. S2), indicating that cAMP-binding to the cNB-A domain, but not to the cNB-B domain, is exclusively concerned with fluorescence modulation of R-FlincA. The apparent *K*_d_ for cGMP was 6.6 μM at pH 7.2, indicating that R-FlincA has a higher selectivity for cAMP rather than cGMP, compared to other indicators (*K*_d_ ratio of cGMP/cAMP is >20 for R-FlincA, 8 for mlCNBD-FRET, 12 for Epac2-camps, 7 for FL2, and 13 for Pink-FL, Supplementary Fig. S3a). However, the sub-μM of cAMP dynamics in cells should be observed carefully if coupled with cGMP dynamics at a high concentration range, since ~7 μM of cGMP, in principle, occupies half of R-FlincA with reduced brightness (60%) compared to the cAMP-bounded form (Supplementary Fig. S3a). The observed p*K*a values of 7.0 (ligand-bound state) and 8.6 (ligand-free state, Supplementary Fig. S3b) are similar to those of other mApple-based indicators^11,27,32,34^. Thus, increased D.R. of R-FlincA has been harnessed within a weakly acidic to neutral pH range (pH 5.5–7.5 for >½ of the maximum D.R.) and the possible pH-dependent signal change should be carefully checked by the parallel control experiments.

### Increased detection sensitivity of R-FlincA

**Figure 2:**
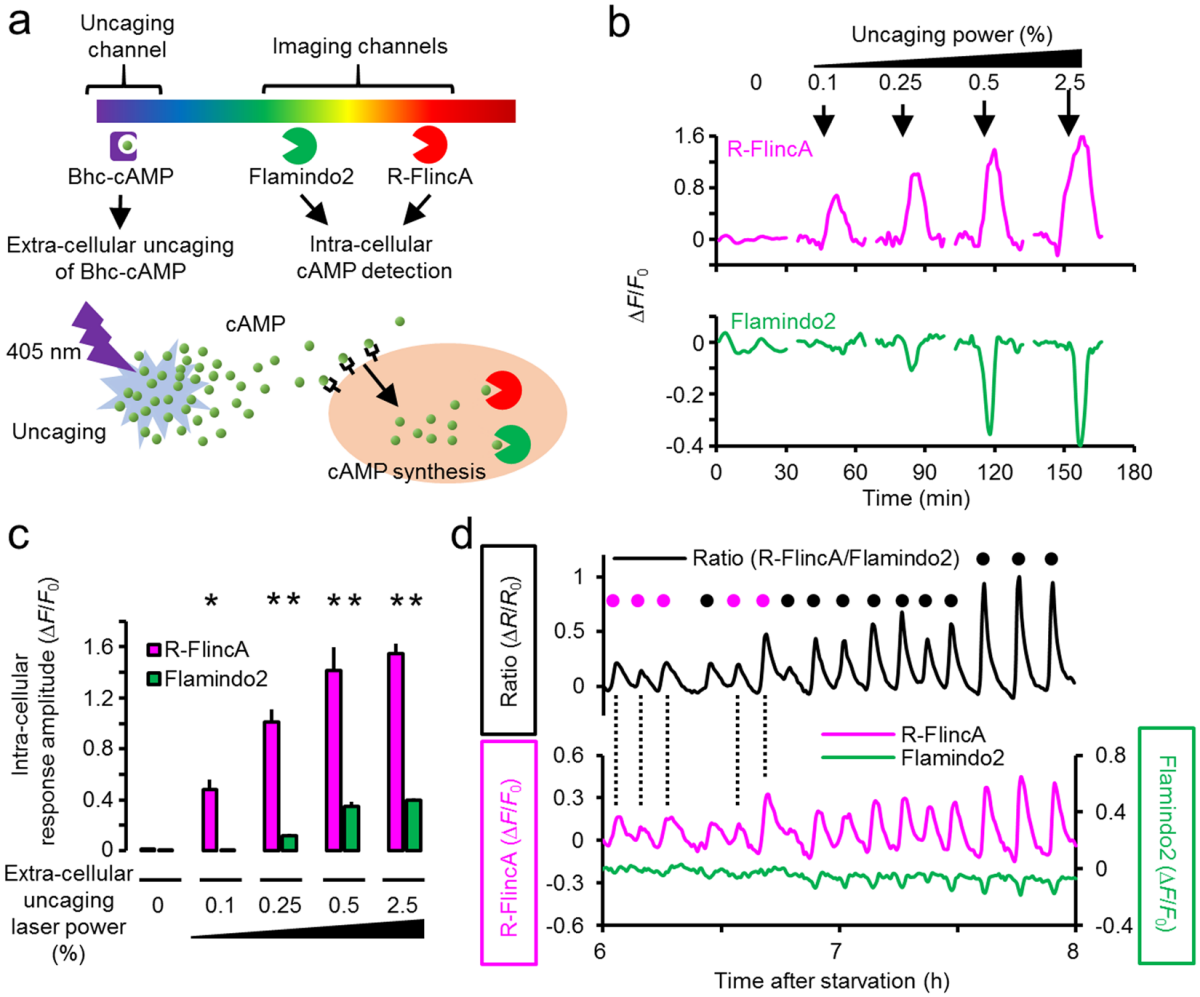
Increased sensitivity of R-FlincA. (**a**) Experimental design for dual-colour cAMP imaging in combination with extra-cellular uncaging of Bhc-cAMP^37^. (**b**) Representative signal changes for R-FlincA and Flamindo2 in a single *D*. *discoideum* cell. Arrows indicate the timing of uncaging (405 nm, 6.7 μsec) with increasing power. (**c**) Signal changes of R-FlincA and Flamindo2 (absolute values of Δ*F*/*F*_0_) in response to extra-cellularly supplied cAMP with increasing uncaging power. Bars represent +SEM, N = 10 cells for laser power 0, 0.1, and 0.25%; N = 5 for 0.5%; N = 15 for 2.5%. Asterisks represent significant differences compared to the control group (0%) at *p* < 0.05 (Student’s t test with two-tailed). (**d**) Spontaneous cAMP dynamics in the population of *D*. *discoideum* cells, detected by the ratiometry of R-FlincA and Flamindo2. The small pulse amplitude at early development (magenta circles) is detected with R-FlincA, but not with Flamindo2. Black circles are the large cAMP pulses detected by both R-FlincA and Flamindo2. *F*_0_ and *R*_0_ is the fluorescence intensity and ratio value at 6 h, respectively. Ratio values were obtained from the pixel-by-pixel calculation. The averaged data in 0.08 mm^2^ containing ~100 cells.

To demonstrate the increased affinity of R-FlincA, we performed comparative cAMP imaging using Flamindo2, an OFF-type yellow fluorescent cAMP indicator, having one order of magnitude lower *K*_d_ value^26^ (*K*_d_ = 3.2 μM, Fig. 1e) than that of R-FlincA. Here, we utilized *D*. *discoideum* cells, which display the intra-cellular cAMP pulse when stimulated by extra-cellular cAMP^35,36^. To observe the intra-cellular cAMP response that displays a dose-dependency on extra-cellular cAMP^35^, we set up triple-channel experiments on a confocal microscope system, wherein FL2 and R-FlincA were imaged using green and red channels, respectively, and the violet channel was used for flash-photolysis of the caged cAMP compound^37^ (Bhc-cAMP) in the extra-cellular space (Fig. 2a). In this experimental setting, extra-cellular cAMP concentration increases proportionally to the power of the uncaging laser, while the resulting cellular cAMP responses are expected to increase in a non-linear manner^35^, since the latter is combinatorially regulated by the sensitivity and gain of the signalling network regulating cAMP synthesis, in addition to input strength. When we observed the cellular cAMP responses at the high-power uncaging, reciprocal changes in the fluorescence intensity were detected by both R-FlincA and FL2, the former has 4-fold larger signal change (Fig. 2b). At the low-power uncaging, the fluorescence signal change was solely detected by R-FlincA, but not by FL2 (Fig. 2b). Simultaneously with the increase in the power of the uncaging laser, the response amplitude of R-FlincA increased significantly, compared to that of FL2 (Fig. 2c), indicating the superiority of R-FlincA to EPAC-based 1-FP indicators in detecting changes in cellular cAMP levels ([cAMP]_in_) induced by the extra-cellular cAMP. The unique detection capability of R-FlincA was further demonstrated by detecting faint pulses of [cAMP]_in_ associated with spontaneous signalling activities not but the artificially induced responses, which is characteristic of the onset dynamics in spontaneous cAMP signalling at early developmental stage (pulses with magenta circles, Fig. 2d).

### Triple-function imaging of cAMP, Ca^2+^, and ATP

**Figure 3:**
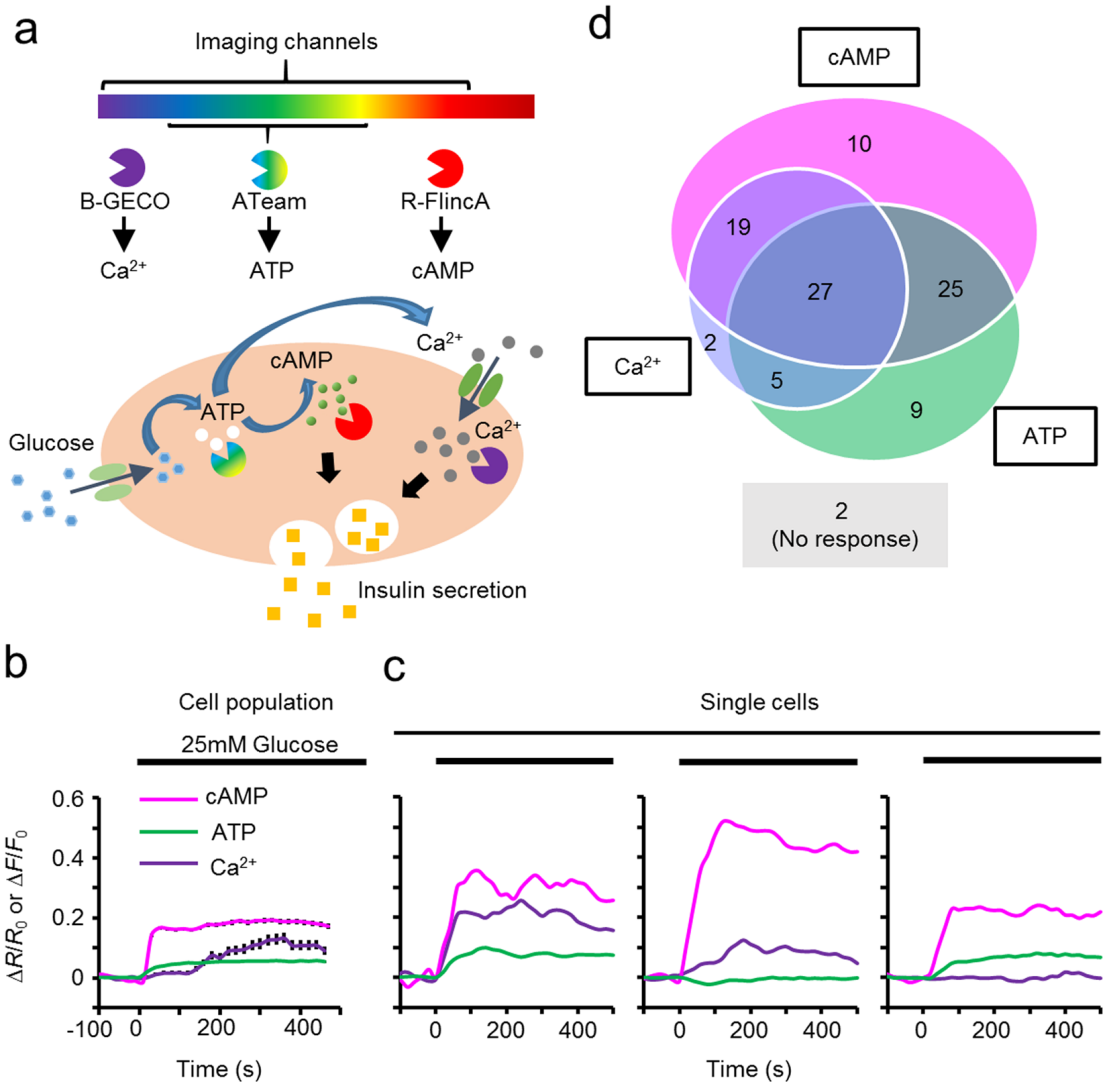
Triple-function imaging of cAMP, Ca^2+^, and ATP. (**a**) Schematic illustration of the triple-function/four-colour imaging in glucose-responsive MIN6 cells. (**b**,**c**) Fluorescence signal changes in B-GECO1, ATeam1.03, and R-FlincA in response to glucose. Averaged traces of a population of MIN6 cells Bars represent ± SEM (N = 99 cells). (**b**) or individual cells (**c**). (**d**) A Venn diagram showing the heterogeneous activation of second messengers. Values are percentages of responding cells out of the total cells (N_total_ = 99 cells).

We further explored the advantages of R-FlincA as a red indicator, as its spectral separation from existing FRET- and 1-FP-based indicators (blue to yellow hue) should allow multi-channel imaging of different signalling events. We, thus, simultaneously observed the dynamics of cellular cAMP, Ca^2+^, and adenosine 5′-triphosphate (ATP), all of which are involved in the glucose-induced insulin secretion of the pancreatic β-cell line MIN6^38–40^ (Fig. 3a). Using the lentiviral vector, we expressed R-FlincA in MIN6 cells, together with B-GECO1^11^ and ATeam1.03^41^, a BFP-based Ca^2+^ and a FRET-based ATP indicator, respectively (Fig. 3a). Triple-function/quadruple-channel imaging (blue, cyan, yellow, and red emission) in the population of MIN6 cells revealed a transient increase in cellular cAMP, Ca^2+^, and ATP ([cAMP]_in_, [Ca^2+^]_in_, and [ATP]_in_) levels, which peaked in a few minutes after stimulation by 25 mM glucose^39,40^ (Fig. 3b). Interestingly, single cell analysis revealed functional heterogeneity in the dynamics of [cAMP]_in_, [Ca^2+^]_in_, and [ATP]_in_ (Fig. 3c). For example, only 30% of the cells were positive for cAMP/Ca^2+^/ATP (Fig. 3c, left), and 2% cells did not show any of the responses. Some were positive for a single response, cAMP (10%), Ca^2+^ (2%), or ATP (10%), while others were positive for two of the three cAMP/Ca^2+^/ATP responses (Fig. 3c, middle/right and Fig. 3d). As MIN6 cells are known to display cell-to-cell heterogeneity in insulin secretion^40,42^, distinct recruitment of these signalling pathways might underlie heterogeneous insulin secretion, which would be suitably addressed by quadruple-function imaging, including exocytotic activity in future analyses.

### Simultaneous imaging of cellular and environmental cAMP

**Figure 4:**
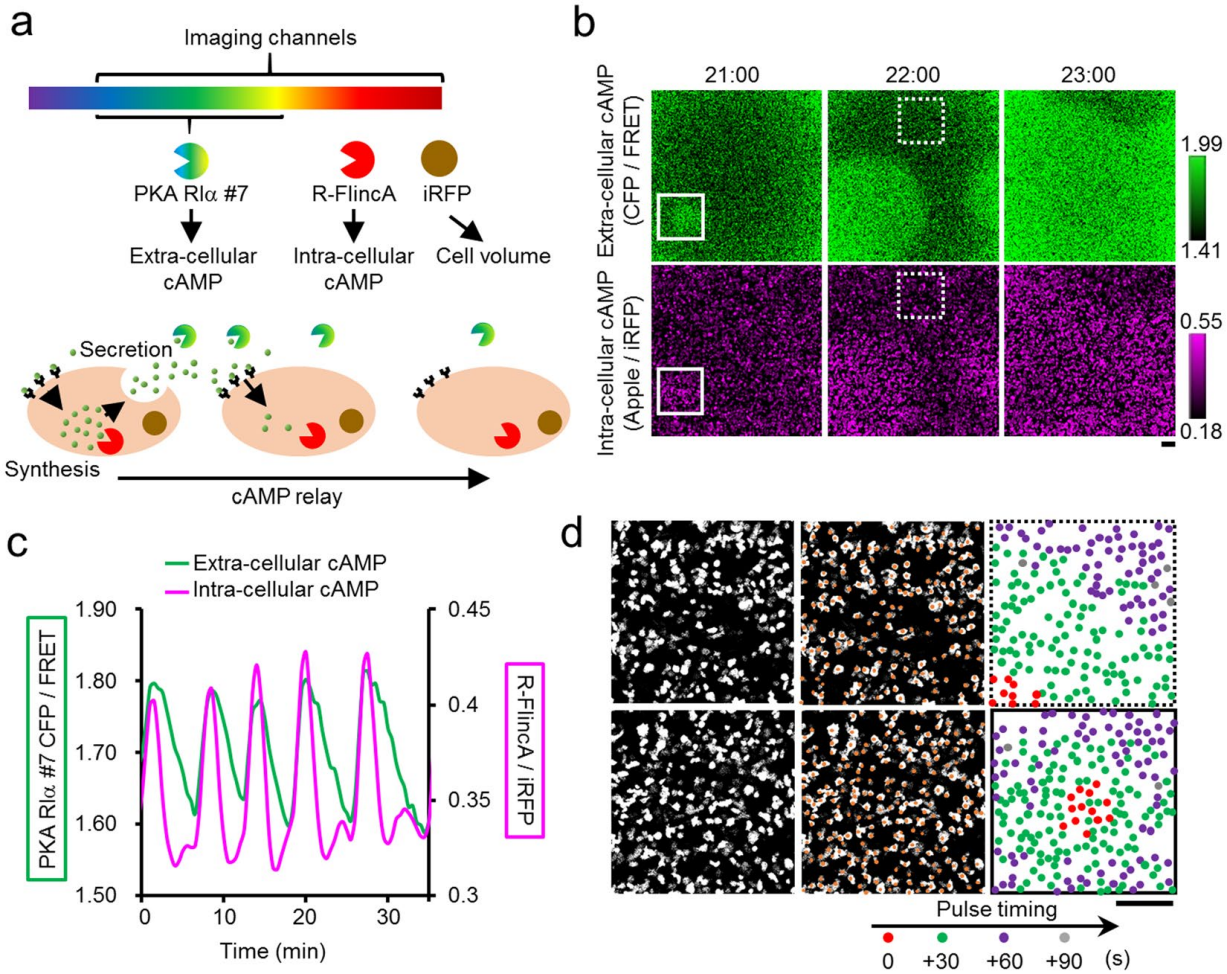
Simultaneous imaging of cellular and environmental cAMP. (**a**) Intra-cellular synthesis and extra-cellular relay of cAMP in a population of *D*. *discoideum* cells, detected by R-FlincA and PKA RIα #7, respectively. Cytoplasmically expressed iRFP was the volume marker for correcting the motion artefact noise in R-FlincA signals. (**b**) The spatio-temporal dynamics of the environmental (top) and cellular (bottom) cAMP in a population of synchronously oscillating *D*. *discoideum* cells. Bar, 100 μm. (**c**) The population-averaged time courses of the [cAMP]_in_ and [cAMP]_ex_ in the solid inset in (**b**). (**d**) Pulse timing at the cell resolution in a wave receiving area (top, dashed inset in (**b**) and the centre of the *de novo* cAMP wave (bottom, solid inset in (**b**). Circles indicate the positions of individual cells. Colours discriminate the pulse timing. Bar, 100 μm. See also Supplementary Video S1.

The utility of R-FlincA was finally demonstrated by simultaneous imaging of cellular and environmental signalling. The signalling dynamics in the microenvironment of cellular population are emerging research targets^43,44^, for which a deeper understanding of the signalling interplay between the cell and its environment is crucial. The population of *D*. *discoideum* cells is a suitable model for cellular and environmental imaging, as these cells self-organize an inter-cellular signalling wave with a sub-mm wavelength, which is achieved by cellular synthesis and extra-cellular relay of cAMP^36,45^. As shown in Fig. 2, these cells synthesize cAMP intra-cellularly upon stimulation by extra-cellular cAMP. Concomitantly, well-developed cells release cAMP into the extra-cellular space. Eventually, the spatially extended chain reaction becomes an outward propagating wave at the cell population level. To simultaneously observe extra-cellular and intra-cellular cAMP dynamics, we utilized PKA RIα #7, an ultra-sensitive FRET-based indicator^23^ (*K*_d_ = 37 nM, CFP-YFP FRET) and R-FlincA, respectively (Fig. 4a). Purified PKA RIα #7 was added to the culture medium containing a population of chemotacting *D*. *discoideum* cells, expressing R-FlincA and iRFP^46^. The latter was utilized as a volume marker correcting the motion artefact noise (Fig. 4a). As previously reported^23^, 6–7 min intervals of [cAMP]_ex_ oscillation were clearly observed with an increase in the FRET ratio of PKA RIα #7 (Fig. 4b top panels, 4c, and Supplementary Video S1). The [cAMP]_in_ in the cell population oscillated with a similar spatio-temporal pattern with [cAMP]_ex_ (Fig. 4b bottom panels, 4c, and Supplementary Video S1). The spatio-temporal mapping of the pulse timing at single-cell resolution showed that the propagating cAMP wave from left-bottom to right-upper corner (dashed inset in Fig. 4b) was achieved by the cAMP relay between the cells (Fig. 4d, top-right). In addition to such a stably propagating wave dominating this fully developed culture, we occasionally observed a *de novo* wave centre (solid inset in Fig. 4b). When this wave initiation region was investigated, the cAMP wave was triggered by a synchronized pulse of 13 cells, localized within 100 × 100 μm^2^ (red circles in Fig. 4d bottom-right), suggesting that the *de novo* cAMP wave initiation would be a collective dynamics of a cluster of cells, not a triggered behaviour by a single cell in the fully developed *D*. *discoideum* population, as reported previously^35^.

## Discussion

In this study, we reported the successful development of R-FlincA whose high-affinity (*K*_d_ = 0.3 μM) and large D.R. (860% at pH 7.2) collectively improves the detection sensitivity of cellular cAMP dynamics. The in-cell brightness of R-FlincA, being 3.5-fold higher than that of Pink-FL, is another advantage in investigating cellular cAMP dynamics in a low concentration range^28,29^. This should be explained on the basis of the increased affinity of R-FlincA and/or increased molecular brightness. Although we could not determine the absolute brightness of R-FlincA molecules in *in vitro*, the maximum brightness of R-FlincA was ~1/4 the parental RFP (mCherry, Supplementary Fig. S4) in a cell-based assay. Pink-FL shows a similar brightness at cAMP-saturated conditions *in vitro*^27^ (~1/5 of mCherry); nonetheless, concentrations of cAMP (*K*_d_ = 7.2 μM, Pink-FL) significantly beyond that producible by the cells is needed. Thus, the increased in-cell brightness of R-FlincA is primarily attributed to its high affinity, which allows for complete harnessing of its expanded D.R. in detecting cellular cAMP dynamics. The sub-optimal brightness of both R-FlincA and Pink-FL would limit certain applications such as the sub-cellular cAMP imaging. Specifically, the fluorescence imaging at the miniscule regions including the dendritic spine^47^ and cilia^28^, suffers from low signals, due to the limited expression of indicators. To improve the S/N ratio, greater increases in the molecular brightness of these indicators is needed by the large-scale molecular evolution, as has been performed for the development of R-GECO^11^. pH-sensitivity and lower quantitativeness are other drawbacks of mApple-based 1-FP indicators; thus, the selection of appropriate cAMP indicators is needed depending on the experimental context; the FRET indicator facilitates reporting of cAMP dynamics with higher brightness, lower pH-sensitivity, and higher quantitativeness. 1-FP indicators including R-FlincA are advantageous in reporting cAMP dynamics with greater changes in signal intensity and in multi-channel applications.

We emphasize that the usability of R-FlincA can be maximized by the combinatorial application with an existing cAMP indicator. The ratiometric imaging of R-FlincA and FL2, an OFF-type yellow indicator^26^ further expands the D.R. (Fig. 2d). Also, their reciprocal signal changes cancel motion artefact noise^48^, which is useful for the imaging of actively locomoting cells, such as leukocytes^49^, and of *D*. *discoideum*^35^. It was not demonstrated through an experiment, we believe R-FlincA would facilitate *in vivo* cAMP imaging. cAMP dynamics, associated with spontaneous cellular activities *in vivo* not but induced dynamics by artificially applied strong stimuli (*e*.*g*., FSK), are a suitable target for R-FlincA. Its increased in-cell brightness would make the true signals more visible under high-background conditions, which suffer from auto-fluorescence and light scattering. The *in vivo* specific motion artefact noises coupled with a heartbeat and respiration would be eliminated if FL2 was co-imaged with R-FlincA. Together with the proof-of-concept demonstrations for multi-colour and multi-function imaging (Figs 2–4), including the combinatorial application with photo manipulation (Fig. 2), R-FlincA will pave the way for a deeper understanding of cAMP dynamics in various fields of life science research, such as neuroscience and developmental biology.

## Methods

### Construction of fluorescent indicators

Red fluorescent cAMP indicators were constructed as follows: cAMP-binding motifs of human PKA RIα (a.a. 93–246 or 93–381, GenBank Accession Number NM_002734.4) were PCR amplified and inserted into *Bam*HI and *EcoR*I restriction sites of pcDNA4/HisMax B (Life Technologies). Subsequently, cpmApple of R-GECO1.2^32^ (a.a. 25–268 in R-GECO1.2 numbering, GenBank Accession Number KF186685.1) was inserted at different positions of the cAMP-binding motifs using In-Fusion HD Cloning Kits (TaKaRa). Mutations in indicator variants were introduced via site directed mutagenesis. Pink-FL was constructed as previously described^27^. Lentiviral vectors expressing R-FlincA, B-GECO1 or ATeam1.03-nD/nA were constructed by inserting these cDNAs into *Bam*HI and *EcoR*I restriction sites of the FUGW vector. A series of packaging plasmids, including FUGW, psPAX2, and pMD2.G were obtained from Addgene. Dual-expression vectors for *D*. *discoideum* cells were constructed as follows: The cDNAs of R-FlincA, FL2, and iRFP^46^ whose codon usage was optimized for *D*. *discoideum* were obtained by a gene synthesis service (Eurofins Genomics), and cloned into an extrachromosomal vector, pDM304^50^. The expression units for FL2 and iRFP were further inserted into the NgoMIV site of pDM304_R-FlincA, yielding pDM304_R-FlincA/FL2 and pDM304_R-FlincA/iRFP. Bi-cistronic vectors expressing R-FlincA or Pink-FL with ECFP were constructed by inserting ECFP cDNA, linked with the sequence encoding the 2A-peptide of porcine teschovirus-1^51^ (P2A), into the end of the cAMP indicators using In-Fusion HD Cloning Kits.

### Cell culture, transfection, and lentivirus production

293 T (RIKEN) and MIN6 cells (kindly provided by Dr. Tamaki at Tokushima University) were maintained in DMEM and RPMI-1640 media (Wako), respectively. Both media were supplemented with 10% FBS, 4 mM L-glutamine (Wako), and 1 mM sodium pyruvate (Wako) at 37 °C. To perform the cell-based indicator screening, 293 T cells were transfected with the expression vectors encoding the cAMP indicators using Lipofectamine 2000 (Life Technologies), and cultured at 32 °C^27^. After 18–24 hours, the culture medium was replaced with Hanks’ buffered saline for imaging. To facilitate the multi-function imaging in MIN6 cells, a high-efficiency gene delivery by lentivirus was utilized. Viral particles were produced in 293 T cells by transfection of the FUGW lentivector together with helper plasmids, psPAX2 and pMD2.G^52^, harvested 48 hours after transfection, and infected to MIN6 cells and cultured at 37 °C. After 4 days, the culture medium of MIN6 cells was replaced with KRBH buffer (2.8 mM Glucose, 129.4 mM NaCl, 5.2 mM KCL, 2.7 mM CaCl_2_, 1.3 mM KH_2_PO4, 1.3 mM MgSO_4_, 24.8 mM NaHCO_3_, and 10 mM HEPES at pH 7.4) for imaging. The axenic strain of *D*. *discoideum* cells (Ax2) was cultured and transformed, as described elsewhere^53,54^. To allow self-organized chemotaxis, cells starved for 4 hours in the development buffer were plated on a glass-bottomed dish (Iwaki). For iRFP imaging, 25 μM biliverdin (Sigma) was added to the culture medium 2 hours before imaging to induce iRFP fluorescence^46^.

### Protein expression and *in vitro* spectroscopy

As the purification of R-FlincA from *E*. *coli* and mammalian cells was not successful, we expressed N-terminal polyhistidine-tagged R-FlincA in *D*. *discoideum* cells cultured in HL5 medium at 22 °C. Cells (8 × 10^8^) were starved in the development buffer (5 mM Na_2_HPO_4_, 5 mM KH_2_PO_4_, 1 mM CaCl_2_, and 2 mM MgCl_2_ at pH 6.4) for 12 hours, and then lysed using n-octyl-1-thio-β-D-glucopyranoside. The recombinant indicator was purified using a Ni-NTA column (Wako), followed by buffer exchange to Hepes buffer (pH 7.4) using a Microsep^TM^ advance centrifugal device 30 K MWCO (PALL). For unknown reasons, the amount of purified R-FlincA was not enough for the absorbance measurements, but it was sufficient for the measurement of excitation and emission spectra. Emission spectra (550–700 nm with a 5-nm bandwidth) upon 530 nm excitation (Xenon, 150 W) and the excitation spectra (450–590 nm) with 635 nm emission were measured using a fluorescence spectrophotometer (F-4500, Hitachi). pH titrations were performed by using a series of buffers prepared with pHs ranging from 4 to 11 as described^8^.

### Imaging and uncaging

Live cell images were captured using an inverted confocal microscope (Nikon A1R, Nikon), equipped with PlanApo20× (0.45 N.A., Nikon), a 405-nm diode laser (36 mW, Melles Griot) for B-GECO1 and uncaging of Bhc-cAMP, a 458-nm Sapphire laser (75 mW, Coherent) for ECFP, ATeam1.03 and PKA RIα #7, a 488- and 561-nm DPSS laser (20 mW and 25 mW, Melles Griot) for FL2 and R-FlincA and Pink-FL, respectively, and a 640-nm diode laser (40 mW, Coherent) for iRFP. Band-pass emission filters were used as follows: 425–475 nm (B-GECO1), 465–500 nm (ECFP, ATeam1.03, and PKA RIα #7), 500–550 nm (FL2), 525–555 nm (ATeam1.03 and PKA RIα #7), 570–620 nm (R-FlincA and Pink-FL), and 663–738 nm (iRFP). The above-mentioned hardware was controlled using the Nikon NIS-Elements software (Nikon), and the image processing was performed with Fiji software (http://fiji.sc/Fiji). Image acquisitions were performed at room temperature for 293T and *D*. *discoideum* cells and at 37 °C for MIN6 cells. For photolytic release (uncaging) of cAMP or environmental cAMP imaging, cell-impermeant Bhc-cAMP^37^ or recombinant PKA RIα #7^23^ was added to the medium, containing 12 hours- or 4 hours-starved *D*. *discoideum* cells (final concentrations of 10 μM or ~100 nM), respectively. Uncaging was performed by a single shot of the 405-nm laser with 0.1–2.5% power for 6.7 μsec. A square area of 2.3 × 2.3 μm^2^, about 100 μm away from the cell, was irradiated.

### Data availability

The data used in this study are available on reasonable request.

## Electronic supplementary material

Supplementary Information

Supplementary Video S1

## References

[1] Kandel ER (2012). The molecular biology of memory: cAMP, PKA, CRE, CREB-1, CREB-2, and CPEB. Mol. Brain.

[2] Mosenden R, Taskén K (2011). Cyclic AMP-mediated immune regulation — Overview of mechanisms of action in T cells. Cell. Signal..

[3] Seino S, Takahashi H, Fujimoto W, Shibasaki T (2009). Roles of cAMP signalling in insulin granule exocytosis. Diabetes Obes. Metab..

[4] Lissandron V, Zaccolo M (2006). Compartmentalized cAMP/PKA signalling regulates cardiac excitation–contraction coupling. J. Muscle Res. Cell Motil..

[5] Miyawaki A (1997). Fluorescent indicators for Ca^2+^ based on green fluorescent proteins and calmodulin. Nature.

[6] Nagai T, Yamada S, Tominaga T, Ichikawa M, Miyawaki A (2004). Expanded dynamic range of fluorescent indicators for Ca^2+^ by circularly permuted yellow fluorescent proteins. Proc. Natl. Acad. Sci. USA.

[7] Nagai T, Sawano A, Park ES, Miyawaki A (2001). Circularly permuted green fluorescent proteins engineered to sense Ca^2+^. Proc. Natl. Acad. Sci..

[8] Baird GS, Zacharias DA, Tsien RY (1999). Circular permutation and receptor insertion within green fluorescent proteins. Proc. Natl. Acad. Sci..

[9] Nakai J, Ohkura M, Imoto K (2001). A high signal-to-noise Ca^2+^ probe composed of a single green fluorescent protein. Nat. Biotechnol..

[10] Tian L (2009). Imaging neural activity in worms, flies and mice with improved GCaMP calcium indicators. Nat. Methods.

[11] Zhao Y (2011). An Expanded Palette of Genetically Encoded Ca^2+^ Indicators. Science.

[12] Chen T-W (2013). Ultrasensitive fluorescent proteins for imaging neuronal activity. Nature.

[13] Jiang JY, Falcone JL, Curci S, Hofer AM (2017). Interrogating cyclic AMP signaling using optical approaches. Cell Calcium.

[14] Patel, N. & Gold, M. G. The genetically encoded tool set for investigating cAMP: more than the sum of its parts. *Front*. *Pharmacol*. **6**, (2015). 10.3389/fphar.2015.00164 PMC4526808 26300778

[15] Adams SR, Harootunian AT, Buechler YJ, Taylor SS, Tsien RY (1991). Fluorescence ratio imaging of cyclic AMP in single cells. Nature.

[16] Zaccolo M (2000). A genetically encoded, fluorescent indicator for cyclic AMP in living cells. Nat. Cell Biol..

[17] Zaccolo M, Pozzan T (2002). Discrete Microdomains with High Concentration of cAMP in Stimulated Rat Neonatal Cardiac Myocytes. Science.

[18] Surdo NC (2017). FRET biosensor uncovers cAMP nano-domains at β-adrenergic targets that dictate precise tuning of cardiac contractility. Nat. Commun..

[19] Mongillo M (2004). Fluorescence Resonance Energy Transfer–Based Analysis of cAMP Dynamics in Live Neonatal Rat Cardiac Myocytes Reveals Distinct Functions of Compartmentalized Phosphodiesterases. Circ. Res..

[20] DiPilato LM, Cheng X, Zhang J (2004). Fluorescent indicators of cAMP and Epac activation reveal differential dynamics of cAMP signaling within discrete subcellular compartments. Proc. Natl. Acad. Sci. USA.

[21] Nikolaev VO, Bünemann M, Hein L, Hannawacker A, Lohse MJ (2004). Novel Single Chain cAMP Sensors for Receptor-induced Signal Propagation. J. Biol. Chem..

[22] Klarenbeek J, Goedhart J, Batenburg A, Van, Groenewald D, Jalink K (2015). Fourth-Generation Epac-Based FRET Sensors for cAMP Feature Exceptional Brightness, Photostability and Dynamic Range: Characterization of Dedicated Sensors for FLIM, for Ratiometry and with High Affinity. PLoS One.

[23] Ohta Y (2016). Nontrivial Effect of the Color-Exchange of a Donor/Acceptor Pair in the Engineering of Förster Resonance Energy Transfer (FRET)-Based Indicators. ACS Chem. Biol..

[24] DiPilato LM, Zhang J (2009). The role of membrane microdomains in shaping β2-adrenergic receptor-mediated cAMP dynamics. Mol. Biosyst..

[25] Smith FD (2017). Local protein kinase A action proceeds through intact holoenzymes. Science.

[26] Odaka H, Arai S, Inoue T, Kitaguchi T (2014). Genetically-Encoded Yellow Fluorescent cAMP Indicator with an Expanded Dynamic Range for Dual-Color Imaging. PLoS One.

[27] Harada K (2017). Red fluorescent protein-based cAMP indicator applicable to optogenetics and *in vivo* imaging. Sci. Rep..

[28] Mukherjee S (2016). A novel biosensor to study cAMP dynamics in cilia and flagella. eLife.

[29] Bacskai BJ (1993). Spatially resolved dynamics of cAMP and protein kinase A subunits in *Aplysia* sensory neurons. Science.

[30] Bubis J, Neitzel JJ, Saraswat LD, Taylor SS (1988). A point mutation abolishes binding of cAMP to site A in the regulatory subunit of cAMP-dependent protein kinase. J. Biol. Chem..

[31] de Rooij J (2000). Mechanism of Regulation of the Epac Family of cAMP-dependent RapGEFs. J. Biol. Chem..

[32] Wu J (2013). Improved Orange and Red Ca^2+^ Indicators and Photophysical Considerations for Optogenetic Applications. ACS Chem. Neurosci..

[33] Cànaves JM, Leon DA, Taylor SS (2000). Consequences of cAMP-Binding Site Mutations on the Structural Stability of the Type I Regulatory Subunit of cAMP-Dependent Protein Kinase. Biochemistry (Mosc.).

[34] Ermakova YG (2014). Red fluorescent genetically encoded indicator for intracellular hydrogen peroxide. Nat. Commun..

[35] Gregor T, Fujimoto K, Masaki N, Sawai S (2010). The Onset of Collective Behavior in Social Amoebae. Science.

[36] Loomis WF (2014). Cell signaling during development of *Dictyostelium*. Dev. Biol..

[37] Furuta T (2004). Bhc-cNMPs as either Water-Soluble or Membrane-Permeant Photoreleasable Cyclic Nucleotides for both One- and Two-Photon Excitation. ChemBioChem.

[38] Vega-Monroy, M.-L. L. de la & Fernandez-Mejia, C. Beta-Cell Function and Failure in Type 1 Diabetes in *Type* 1 *Diabetes - Pathogenesis*, *Genetics and Immunotherapy* (ed. Wagner, D.) 93–116 (InTech, 2011).

[39] Tanaka T (2014). Glucose-stimulated single pancreatic islets sustain increased cytosolic ATP levels during initial Ca^2+^ influx and subsequent Ca^2+^ oscillations. J. Biol. Chem..

[40] Landa LR (2005). Interplay of Ca^2+^ and cAMP Signaling in the Insulin-secreting MIN6 β-Cell Line. J. Biol. Chem..

[41] Kotera I, Iwasaki T, Imamura H, Noji H, Nagai T (2010). Reversible Dimerization of *Aequorea victoria* Fluorescent Proteins Increases the Dynamic Range of FRET-Based Indicators. ACS Chem. Biol..

[42] Hauge-Evans AC, Squires PE, Persaud SJ, Jones PM (1999). Pancreatic beta-cell-to-beta-cell interactions are required for integrated responses to nutrient stimuli: enhanced Ca^2+^ and insulin secretory responses of MIN6 pseudoislets. Diabetes.

[43] Joyce JA, Pollard JW (2009). Microenvironmental regulation of metastasis. Nat. Rev. Cancer.

[44] Gajewski TF, Schreiber H, Fu Y-X (2013). Innate and adaptive immune cells in the tumor microenvironment. Nat. Immunol..

[45] Dormann D, Vasiev B, Weijer CJ (1998). Propagating waves control *Dictyostelium discoideum* morphogenesis. Biophys. Chem..

[46] Filonov GS (2011). Bright and stable near-infrared fluorescent protein for *in vivo* imaging. Nat. Biotechnol..

[47] Ji Y, Pang PT, Feng L, Lu B (2005). Cyclic AMP controls BDNF-induced TrkB phosphorylation and dendritic spine formation in mature hippocampal neurons. Nat. Neurosci..

[48] Kanemaru K (2014). *In Vivo* Visualization of Subtle, Transient, and Local Activity of Astrocytes Using an Ultrasensitive Ca^2+^ Indicator. Cell Rep..

[49] Grewe SR, Chan SC, Hanifin JM (1982). Elevated leukocyte cyclic AMP—phosphodiesterase in atopic disease: a possible mechanism for cyclic AMP—agonist hyporesponsiveness. J. Allergy Clin. Immunol..

[50] Veltman DM, Akar G, Bosgraaf L, Van Haastert PJM (2009). A new set of small, extrachromosomal expression vectors for *Dictyostelium discoideum*. Plasmid.

[51] Kim JH (2011). High Cleavage Efficiency of a 2A Peptide Derived from Porcine Teschovirus-1 in Human Cell Lines, Zebrafish and Mice. PLoS One.

[52] Lois C, Hong EJ, Pease S, Brown EJ (2002). & Baltimore, D. Germline Transmission and Tissue-Specific Expression of Transgenes Delivered by Lentiviral Vectors. Science.

[53] Gaudet P, Pilcher KE, Fey P, Chisholm RL (2007). Transformation of *Dictyostelium discoideum* with plasmid DNA. Nat. Protoc..

[54] Fey P, Kowal AS, Gaudet P, Pilcher KE, Chisholm RL (2007). Protocols for growth and development of *Dictyostelium discoideum*. Nat. Protoc..

