# Supplementary material for: Red fluorescent cAMP indicator with increased affinity and expanded dynamic range
Source: Sci Rep. 2018 Jan 30;8:1866. doi: 10.1038/s41598-018-20251-1 (PMC5789972; doi:10.1038/s41598-018-20251-1)
Supplement: Supplementary file 1 — Supplementary Information [file 41598_2018_20251_MOESM1_ESM.pdf]

Supplementary Information for:

Red fluorescent cAMP indicator with increased affinity and expanded dynamic range

Yusaku Ohta, Toshiaki Furuta, Takeharu Nagai, and Kazuki Horikawa

Contents

1. Supplementary Figures

2. Legend for Supplementary Video

3. Supplementary Tables

1. Supplementary Figures

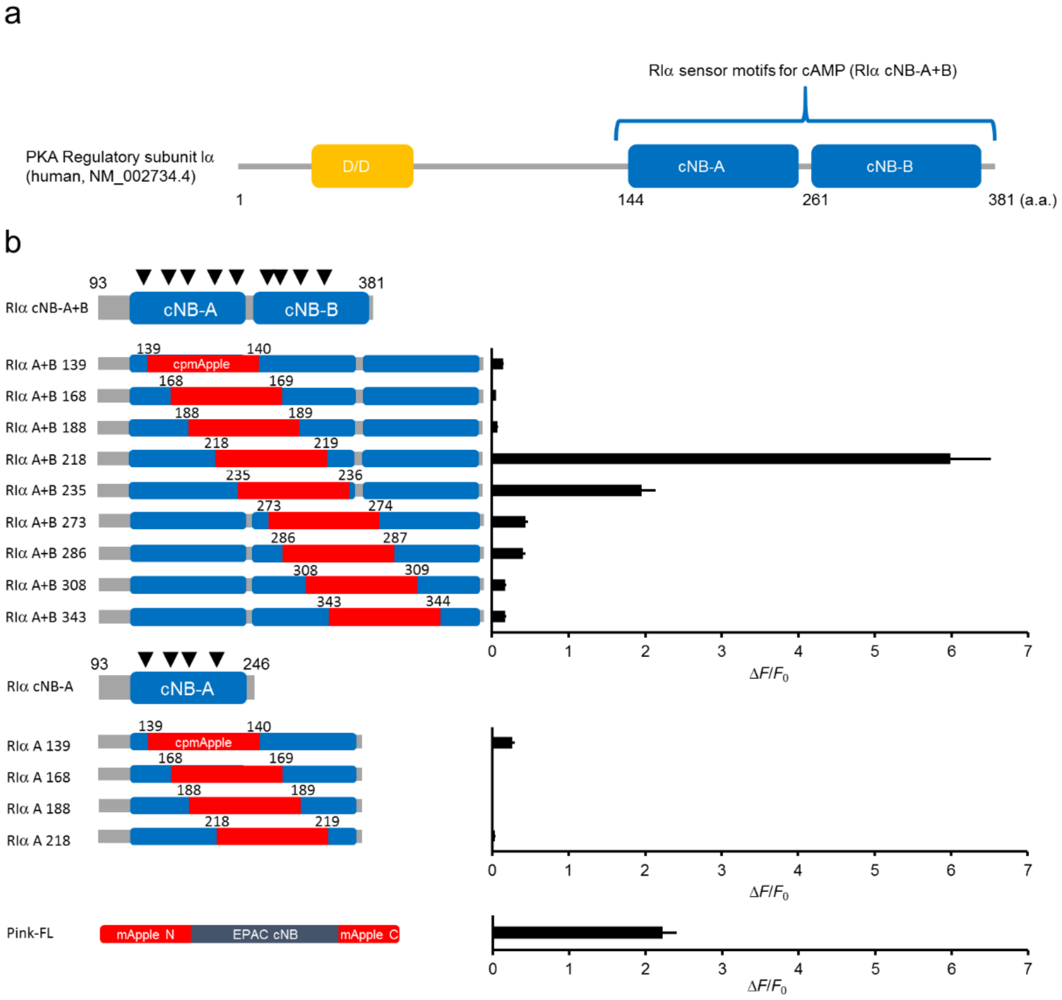

Supplementary Figure S1. Development of an improved cAMP indicator.

(**a**) cAMP-binding motif of PKA RIα utilized in this study. D/D: dimerization/docking domain, cNB-A/B: cyclic nucleotide binding domain. (**b**) The candidate constructs with the cp146mApple insertion at different positions of RIα (left). The response of these constructs in the cell-based assay using 293T cells. The fluorescence signal change ($\Delta F/F_0$) to the forced elevation of cellular cAMP by FSK treatment (right). Bars represent + SEM (N = 20 cells, respectively). The a.a. numbering represents the position in the full-length RIα. Pink-FL was used as a positive control.

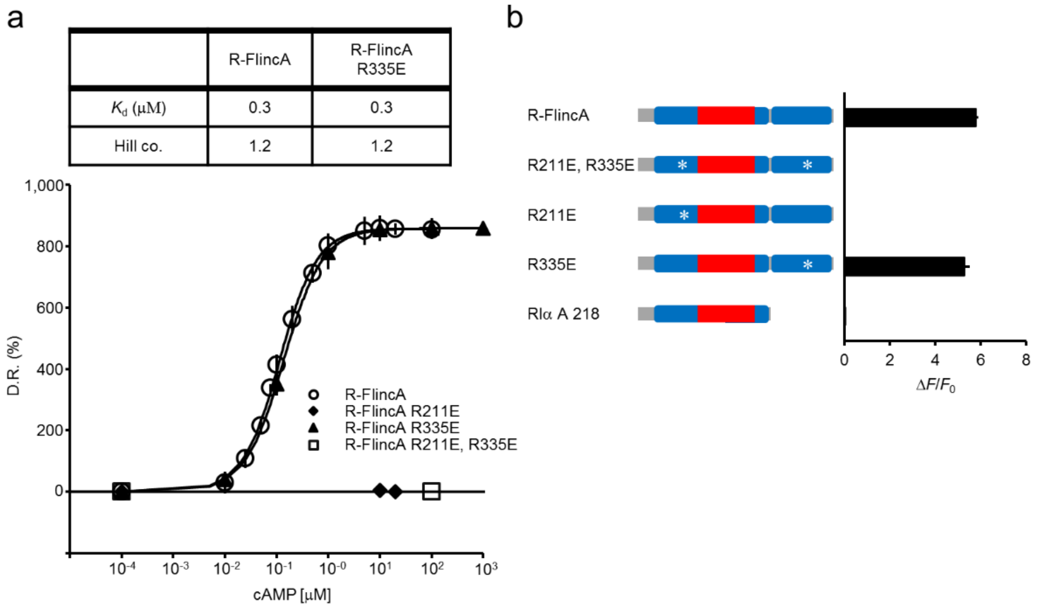

Supplementary Figure S2. The contribution of two-binding sites on the properties of R-FlincA. (**a**) Titration curves for cAMP of R-FlincA variants at pH 7.2. R211E and R335E (in RIα numbering) are the mutations in cNB-A and cNB-B that eliminate cAMP-binding, respectively. For R-FlincA, K_d = 0.30 ± 0.02 (mean ± SEM), Hill coefficient = 1.19 ± 0.06; For R-FlincA R335E, K_d = 0.30 ± 0.04 μM, Hill coefficient = 1.16 ± 0.07. Bars represent ± SEM for triplicate experiments. (**b**) Dynamic range of R-FlincA variants assayed in 293T cells. Bars represent + SEM (N = 20–40 cells) for duplicate experiments. Deletion of cNB-B domain (RIα A 218) abolished the fluorescence increase upon FSK stimulation, indicating that the presence of cNB-B is necessary for increase in the fluorescence intensity of R-FlincA, although a cAMP-binding to cNB-B is not essential. The a.a. numbering represents the position in the full-length RIα.

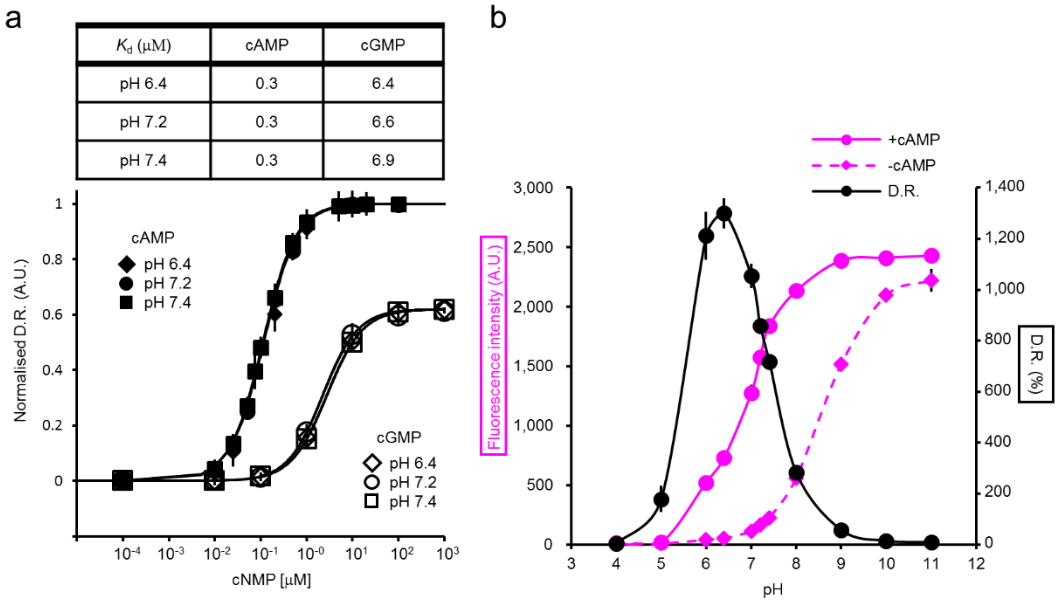

Supplementary Figure S3. Additional *in vitro* properties of R-FlincA.

(**a**) A set of titration curves for cAMP and cGMP at a given pH, the both were normalised by the D.R. of cAMP-bound form (100 μM). Three different pH conditions were tested: pH 7.2 and 7.4 are the physiologically relevant intra-cellular pH. pH 6.4 is the condition yielding the largest D.R. (1,300%, see also Figure S3b). K_ds for cAMP are 0.29 ± 0.02 (mean ± SEM), 0.30 ± 0.02 and 0.30 ± 0.03, at pH 6.4, 7.2 and 7.4, respectively. K_ds for cGMP are 6.36 ± 0.14, 6.61 ± 0.21 and 6.87 ± 0.25 μM, at pH 6.4, 7.2 and 7.4, respectively. Bars represent ± SEM for three independent experiments. (**b**) The fluorescence intensity (left axis) in the presence (solid magenta) or absence (dashed magenta) of 1 mM cAMP, plotted against pH. The dynamic range (right axis) was calculated by dividing the difference (ΔF) between the intensities of the cAMP-bound form and cAMP-free form by that of the cAMP-free form (F_0). Bars represent ± SEM for triplicate experiments.

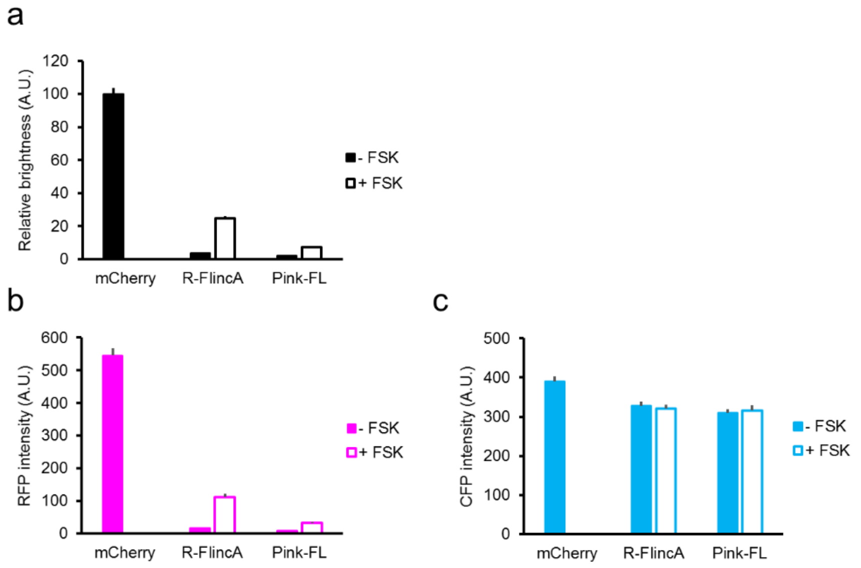

Supplementary Figure S4. In-cell brightness of red cAMP indicators.

(**a**) The intensities of RFPs before and after FSK stimulation were divided by those of bi-cistronically expressed ECFP, then were normalised to the brightness of mCherry (100%). The relative brightness of R-FlincA before and after FSK stimulation were 3.60 ± 0.24 (mean ± SEM) and 24.9 ± 1.03, respectively. Those of Pink-FL before and after FSK stimulation were 2.06 ± 0.22 and 7.21 ± 0.40, respectively. Bars represent + SEM (N = 30, 30, and 40 cells for mCherry, R-FlincA, and Pink-FL) for duplicate experiments. (**b, c**) The raw intensities of RFPs and ECFP were shown in (b) and (c), respectively.

2. Legend for Supplementary Video

Supplementary Video S1 (.mp4)

Extra-cellular and intra-cellular dual imaging by PKA RIα #7 and R-FlincA in the population of *D. discoideum* cells. The left and right panels show FRET ratio images of the culture medium containing PKA RIα #7 and R-FlincA/iRFP ratio images of *D. discoideum* cells, respectively. Bar, 100 μm.

3. Supplementary Tables

Supplementary Table S1. Properties of 1-FP cAMP indicators, sorted by the affinity. Modified from Ref. (13).

cAMP indicator	Dynamic range[a] (%)	K_d (μM)	FP	References
R-FlincA	860[b]	0.3[b]	cp146mApple	This study
Flamindo2	300[c]	3.2[c]	Citrine	Odaka *et al.*[26]
Pink Flamindo	320[c]	7.2[c]	mApple	Harada *et al.*[27]

[a]$(F_{max}-F_{min})/F_{min}$, where F_{max} and F_{min} is the maximum and minimum fluorescence intensity, respectively.

[b]The value obtained by *in vitro* spectroscopy at pH 7.2.

[c]The value obtained by *in vitro* spectroscopy at pH 7.4.

Supplementary Table S2. Properties of selected FRET cAMP indicators, sorted by the affinity. Modified from Ref. (13).

cAMP indicator	Dynamic range[a] (%)	K_d (μM)	Donor & Acceptor combination[f]	References
PKA RIα #7	40[b]	0.04[b]	cp173Venus/ECFP	Ohta *et al.*[23]
mlCNBD-FRET	50[c]	0.07[b]	Citrine/Cerulean	Mukherjee *et al.*[28]
R-CFP, C-YFP	~20[d]	0.3[b]	YFP, CFP	Zaccolo *et al.*[17], and Smith *et al.*[25] Mongillo *et al.*[19]
Epac2-camps	~20[d]	0.9[b]	EYFP/ECFP	Nikolaev *et al.*[21]
Epac-S[H187]	160[c]	~4.0[f]	mTurquoise2/Td-cp173Venus	Klarenbeek *et al.*[22]
CUTie	~20[e]	7.4[c]	EYFP/ECFP	Surdo *et al.*[18]
RII-EBFP, C-S65T	N/A	N/A	EBFP, EGFP	Zaccolo *et al.*[16]
ICUE3	100[c]	N/A	ECFP/cp194Venus	Dipilato *et al.*[24] and Smith *et al.*[25]

[a]$(R_{max}-R_{min})/R_{min}$, where R_{max} and R_{min} is the maximum and minimum emission ratio, respectively.
[b]The value determined by *in vitro* spectroscopy in the referenced study.
[c]The value determined by in-cell observations in the referenced study.
[d]The estimated value (~) from the spectroscopic data in the cited paper.
[e]The estimated value (~) from the in-cell data in the cited paper.
[f]The value (~) of the related construct (Epac-S[H134]) sharing Q270E mutation on EPAC.
[g]The combination of the donor and acceptor with its order.
